# Once-weekly glucagon-like peptide-1 receptor agonists vs dipeptidyl peptidase-4 inhibitors: cardiovascular effects in people with diabetes and cardiovascular disease

**DOI:** 10.1186/s12933-023-02051-8

**Published:** 2023-11-20

**Authors:** Xi Tan, Yuanjie Liang, Jigar R. Rajpura, Larisa Yedigarova, Josh Noone, Lin Xie, Silvio Inzucchi, Adam de Havenon

**Affiliations:** 1 Novo Nordisk Inc., Plainsboro, NJ USA; 2 Department of Endocrinology, Yale University, New Haven, CT USA; 3 Department of Neurology, Center for Brain and Mind Health, Yale School of Medicine, Yale University, 15 York St, New Haven, CT 06510 USA

**Keywords:** Type 2 diabetes, Atherosclerotic cardiovascular disease, Glucagon-like peptide-1 receptor agonists, Stroke, Myocardial infarction, Major adverse cardiovascular events

## Abstract

**Background:**

Glucagon-like peptide-1 receptor agonists (GLP-1 RAs), which have proven cardiovascular benefits, are recommended in people with type 2 diabetes (T2D) and atherosclerotic cardiovascular disease (ASCVD). However, there is limited real-world evidence comparing the effects of once-weekly (OW) GLP-1 RAs and dipeptidyl peptidase-4 inhibitors (DPP-4is). This observational cohort study (1/1/2017–9/30/2021) used data from the Optum Clinformatics^®^ Data Mart to compare time to incident clinical cardiovascular outcomes, health care resource utilization (HCRU), and medical costs in new adult users of OW GLP-1 RAs and DPP-4is with T2D and ASCVD.

**Methods:**

Time to occurrence of ischemic stroke, myocardial infarction (MI), or their composite and ASCVD-related and all-cause HCRU and medical costs were investigated. Baseline characteristics were balanced using inverse probability of treatment weighting. Survival analyses were conducted to compare risks during exposure.

**Results:**

OW GLP-1 RA users (weighted N = 25,287) had 26%, 22%, and 24% lower risk of ischemic stroke, MI, and their composite, respectively, compared with DPP-4i users (weighted N = 39,684; all *P* < 0.01). Compared with DPP-4i users, OW GLP-1 RA users had 25% and 26% lower ASCVD-related and all-cause hospitalization costs, 19% and 23% lower ASCVD-related and all-cause medical costs, 23% and 27% fewer ASCVD-related and all-cause hospitalizations, 13% and 8% fewer ASCVD-related and all-cause outpatient visits, and 8% fewer all-cause ER visits (all *P* < 0.01).

**Conclusions:**

In adults with T2D and ASCVD, OW GLP-1 RAs are associated with reduced stroke and MI risks and ASCVD-related and all-cause HCRU and costs vs DPP-4is.

**Supplementary Information:**

The online version contains supplementary material available at 10.1186/s12933-023-02051-8.

## Introduction

In people with type 2 diabetes (T2D), atherosclerotic cardiovascular disease (ASCVD) is the leading cause of morbidity and mortality and is associated with significant health care utilization and cost [1–4]. Several large cardiovascular outcome trials (CVOTs) have demonstrated that glucose-lowering agents belonging to the glucagon-like peptide-1 receptor agonist (GLP-1 RA) class significantly reduce cardiovascular events vs baseline glucose-lowering therapy in high-risk individuals with T2D [1, 5, 6]. In keeping with these data, diabetes and cardiology guidelines and professional societies recommend a GLP-1 RA with demonstrated cardiovascular benefit for the treatment of individuals with T2D and established or at high risk for ASCVD. This recommendation is independent of baseline or target glycated hemoglobin (HbA1c) levels [1, 5–7].

Dipeptidyl peptidase-4 inhibitors (DPP-4is) are also incretin-based therapies commonly used in clinical practice for people with T2D [8]. While GLP-1 RAs have demonstrated cardiovascular benefits in CVOTs, DPP-4is have not [8]. However, head-to-head trials specifically comparing the effects of these 2 drug classes on cardiovascular outcomes in individuals with T2D and established ASCVD are lacking. It is important to evaluate their cardiovascular benefits in longitudinal, real-world studies to explore the effects of these therapies in larger populations who do not take part in clinical trials and to analyze health care utilization and costs in these populations.

Some evidence has also suggested that the cardiovascular benefits of GLP-1 RAs may not be a class effect [8]. Older generations of daily GLP-1 RAs may be less efficacious in reducing cardiovascular events [8]; therefore, including them in assessments of the entire GLP-1 RA class may generate mixed effects. It is instead important to examine the cardiovascular effectiveness of the newer, once-weekly (OW) generation of GLP-1 RAs, which have been linked to improvements in glycemic control [9, 10], reduced body weight [9, 11], and better adherence/persistence relative to daily injectable GLP-1 RAs [12] and now comprise the vast majority of prescriptions for this class in the US [13]. However, real-world evidence comparing cardiovascular outcomes in US adults with T2D and established ASCVD who initiated a OW GLP-1 RA vs a DPP-4i is limited.

This study aims to compare the time to occurrence of ischemic stroke, MI, and their composite, and ASCVD-related and all-cause health care resource utilization (HCRU) and medical costs in people with T2D and ASCVD who initiated a OW GLP-1RA vs a DPP-4i.

## Methods

### Study design

This was an observational cohort study using the Optum Clinformatics^®^ Data Mart (CDM) database to compare cardiovascular outcomes in adults with T2D and established ASCVD who initiated a OW GLP-1 RA or a DPP-4i. The study period was from January 1, 2017, to September 30, 2021. ASCVD history was examined back to 2001. OW GLP-1 RAs studied included exenatide, dulaglutide, and semaglutide. DPP-4is included sitagliptin, saxagliptin, linagliptin, and alogliptin. Individuals who switched within the same drug class during the follow-up period were included, but those who switched between classes were excluded.

The index date, which was between January 1, 2018, and June 30, 2021, was defined as the prescription date of the index drug (OW GLP-1 RA or DPP-4i). The beginning of the index window was selected to fall immediately after the approval of the newest agent included in this study (once weekly semaglutide, approved for T2D in December 2017). One year prior to the index date constituted the baseline period. Individuals were followed for ≥ 3 months, until censoring due to the earliest of death; end of the study (September 30, 2021); new initiation of a sodium-glucose cotransporter-2 (SGLT-2) inhibitor, DPP-4i (among those in the GLP-1 RA group), or GLP-1 RA (among those in the DPP-4i group); lapse of continuous enrollment; or discontinuation of the index drug for > 60 days. The follow-up period, in which outcomes/end points were assessed, was the interval between the index date and end of follow-up.

### Data source

The Optum CDM contains administrative claims for enrollees of commercial health care plans and Medicare Advantage across the US. The administrative claims include verified, adjudicated, adjusted, and de-identified medical and pharmacy claims. The CDM also includes outpatient laboratory test results from large national laboratory vendors.

### Study population

Individuals included in the study had ≥ 2 separate diagnoses of T2D on different dates during the study period, identified using the *International Classification of Diseases, Tenth Revision, Clinical Modification (ICD-10-CM)* code E11 in the primary or secondary diagnosis positions; ≥ 1 prescription for the index drug; and use of the index drug for ≥ 90 days (with ≤ 60-day gaps). Individuals were ≥ 18 years old on the index date and had a history of ASCVD between 2001 and the index date, identified using the *International Classification of Diseases, Ninth Revision, Clinical Modification (ICD-9-CM)* and *ICD-10-CM* codes in any position (see Additional file 1).

Exclusion criteria included baseline GLP-1 RA or DPP-4i use, missing demographic information (age or sex), or pregnancy or type 1 diabetes at any time during the baseline or follow-up periods.

Detailed baseline characteristics are listed in Table 1 and Additional file 2. As this study used only de-identified patient records and did not involve the collection, use or transmittal of individually identifiable data, institutional review board approval was not required.

**Table 1:** Selected unweighted and weighted key baseline characteristics among adults with T2D and ASCVD

Variable	Category	Unweighted					Weighted				
		DPP-4i (N = 39,858)		OW GLP-1 RA (N = 26,430)		SMD	DPP-4i (N = 39,684)		OW GLP-1 RA (N = 25,287)		SMD
		Mean	SD	Mean	SD		Mean	SD	Mean	SD	
Age, years	–	72.67	9.75	66.53	10.08	0.619*	70.26	10.32	69.68	10.19	0.057
ASCVD, years	–	4.20	3.80	4.07	3.81	0.034	4.14	3.80	4.12	3.78	0.005
T2D, years	–	5.40	4.13	5.42	4.30	0.005	5.34	4.14	5.47	4.24	0.029
CCI score	–	2.71	2.27	2.26	2.08	0.204*	2.53	2.21	2.48	2.19	0.026
DCSI score	–	3.40	2.20	3.14	2.18	0.119*	3.29	2.19	3.26	2.21	0.017
HbA1c^a^	–	8.06	1.68	8.59	1.77	0.312*	8.28	1.68	8.39	1.70	0.066
BMI^b^, kg/m^2^	–	33.07	7.75	37.49	8.41	0.547*	34.91	8.25	35.52	8.38	0.073
Out-of-pocket Rx cost, USD	–	721.87	819.93	878.14	992.05	0.172*	753.72	823.55	814.52	932.05	0.069
No. of ER visits	–	0.76	1.74	0.74	2.00	0.012	0.74	1.76	0.73	1.91	0.006
No. of IP visits	–	0.69	1.64	0.46	1.22	0.163*	0.60	1.49	0.55	1.42	0.033

Variable	Category	n	%	n	%	SMD	n	%	n	%	SMD
Age group	18–44 year	297	0.8	618	2.3	0.130*	539	1.4	367	1.5	0.008
	45–64 year	6928	17.4	9651	36.5	0.442*	9852	24.8	6628	26.2	0.032
	65–79 year	22,570	56.6	13,924	52.7	0.079	21,909	55.2	14,151	56.0	0.015
	≥ 80 year	10,063	25.3	2237	8.5	0.460*	7384	18.6	4141	16.4	0.059
Sex	F	19,953	50.1	12,767	48.3	0.035	19,645	49.5	12,387	49.0	0.010
	M	19,905	49.9	13,663	51.7	0.035	20,039	50.5	12,900	51.0	0.010
Region	0; Northeast	4823	12.1	2491	9.4	0.086	4348	11.0	2656	10.5	0.015
	1; South	21,514	54.0	14,620	55.3	0.027	21,767	54.9	13,981	55.3	0.009
	2; Midwest	6417	16.1	4842	18.3	0.059	6671	16.8	4272	16.9	0.002
	3; West	7082	17.8	4462	16.9	0.023	6874	17.3	4360	17.2	0.002
	4; Unknown	22	0.1	15	0.1	0.001	24	0.1	18	0.1	0.005
Payer type	COM	5111	12.8	7397	28.0	0.383*	7295	18.4	4940	19.5	0.029
	MCR	34,747	87.2	19,033	72.0	0.383*	32,389	81.6	20,347	80.5	0.029
Index year	2018	11,683	29.3	4369	16.5	0.308*	9759	24.6	5970	23.6	0.023
	2019	11,297	28.3	7388	28.0	0.009	11,251	28.4	7192	28.4	0.002
	2020	10,412	26.1	8414	31.8	0.126*	11,172	28.2	7225	28.6	0.009
	2021	6466	16.2	6259	23.7	0.187*	7501	18.9	4900	19.4	0.012
Race/ethnicity	0; White	21,936	55.0	16,479	62.4	0.149*	22,988	57.9	14,866	58.8	0.017
	1; Black	6496	16.3	4256	16.1	0.005	6457	16.3	4137	16.4	0.002
	2; Hispanic	7566	19.0	3886	14.7	0.115*	6838	17.2	4211	16.7	0.015
	3; Asian	1970	4.9	581	2.2	0.148*	1519	3.8	854	3.4	0.024
	4; Unknown	1890	4.7	1228	4.7	0.005	1882	4.7	1220	4.8	0.004
Number of glucose-lowering therapies	0	3937	9.9	1652	6.3	0.134*	3260	8.2	2032	8.0	0.006
	1	16,921	42.5	8905	33.7	0.181*	15,416	38.9	9232	36.5	0.048
	2	14,294	35.9	10,102	38.2	0.049	14,648	36.9	9669	38.2	0.027
	3 +	4706	11.8	5771	21.8	0.271*	6360	16.0	4354	17.2	0.032
Glucose-lowering therapy use	Metformin	28,044	70.4	18,060	68.3	0.044	27,641	69.7	17,630	69.7	0.001
	SU	16,648	41.8	9436	35.7	0.125*	15,893	40.1	10,234	40.5	0.009
	TZD	3010	7.6	2263	8.6	0.037	3216	8.1	2127	8.4	0.011
	SGLT-2	3785	9.5	5435	20.6	0.313*	5599	14.1	3819	15.1	0.028
	Insulin	7946	19.9	11,888	45.0	0.555*	11,792	29.7	8019	31.7	0.043
	AGI	191	0.5	117	0.4	0.005	188	0.5	128	0.5	0.005
	MEG	619	1.6	276	1.0	0.045	538	1.4	333	1.3	0.003
Other medication use	Anticoagulants	6037	15.2	3536	13.4	0.051	5789	14.6	3454	13.7	0.027
	Antihypertensives	36,784	92.3	24,286	91.9	0.015	36,536	92.1	23,207	91.8	0.011
	Antiplatelets	7252	18.2	4822	18.2	0.001	7212	18.2	4693	18.6	0.010
	Misc hyperlipidemic	5656	14.2	4335	16.4	0.061	5921	14.9	3852	15.2	0.009
	PCSK9	146	0.4	228	0.9	0.064	178	0.5	179	0.7	0.034
	Statins	32,277	81.0	21,731	82.2	0.032	32,274	81.3	20,627	81.6	0.006
Other comorbidities	Atrial fibrillation	6669	16.7	3478	13.2	0.100*	6075	15.3	3646	14.4	0.025
	Alcohol use disorder	767	1.9	474	1.8	0.010	781	2.0	435	1.7	0.018
	Anxiety	6681	16.8	5114	19.4	0.067	6959	17.5	4553	18.0	0.012
	Depression	9456	23.7	7145	27.0	0.076	9766	24.6	6551	25.9	0.030
	Hyperlipidemia	35,306	88.6	23,633	89.4	0.027	35,167	88.6	22,539	89.1	0.016
	Hypertension	37,304	93.6	24,534	92.8	0.030	36,990	93.2	23,540	93.1	0.005
	Obesity	14,950	37.5	14,857	56.2	0.382*	17,837	45.0	11,737	46.4	0.029
	Smoking	4598	11.5	3556	13.5	0.058	4998	12.6	3033	12.0	0.018
	Chronic heart failure	9961	25.0	6026	22.8	0.051	9538	24.0	5970	23.6	0.010
	Cancer	4883	12.3	2431	9.2	0.099	4401	11.1	2683	10.6	0.015
	CKD	15,924	40.0	8086	30.6	0.197*	14,403	36.3	8896	35.2	0.023
Type of ASCVD	MI	5840	14.7	3842	14.5	0.003	5767	14.5	3672	14.5	0.000
	Ischemic stroke	6545	16.4	3602	13.6	0.078	6047	15.2	3745	14.8	0.012
	PAD	21,438	53.8	12,565	47.6	0.125*	20,373	51.4	12,827	50.7	0.012
	TIA	5106	12.8	2864	10.8	0.061	4780	12.1	2936	11.6	0.013
	Other atherosclerotic cerebrovascular disease	14,350	36.0	7942	30.1	0.127*	13,444	33.9	8304	32.9	0.022
	Other CHD	26,059	65.4	17,800	67.4	0.042	26,167	66.0	16,837	66.6	0.014

*AGI* alpha-glucosidase inhibitor, *ASCVD* atherosclerotic cardiovascular disease, *BMI* body mass index, *CABG* coronary artery bypass grafting, *CCI* Charlson Comorbidity Index, *CHD* coronary heart disease, *CKD* chronic kidney disease, *COM* commercial, *DCSI* diabetes complication severity index, *DPP-4i* dipeptidyl peptidase-4 inhibitor, *ER* emergency room, *GLP-1 RA* glucagon-like peptide-1 receptor agonist, *HbA1c* glycated hemoglobin, *ICD-10 International Classification of Diseases, Tenth Revision*, *IP* inpatient, *MCR* Medicare, *MEG* meglitinide, *MI* myocardial infarction, OW once-weekly, *PAD* peripheral arterial disease, *PCI* percutaneous coronary intervention, *PCSK9* proprotein convertase subtilisin/kexin type 9, *Rx* prescription, *SGLT-2* sodium-glucose cotransporter 2 [inhibitor], *SMD* standardized mean difference, *SU* sulfonylurea, *T2D* type 2 diabetes, *TIA* transient ischemic attack, *TZD* thiazolidinedione

^*^Indicates significant difference

^a^HbA1c results reflect only those individuals for whom these data were available

^b^BMI results reflect only those individuals for whom these data were available. Continuous BMI values were calculated as the midpoint of the value range for the corresponding BMI code. (For example, a value of 32.5 was used for the *ICD-10* code Z68.32, which includes BMI 32.0–32.9.)

### Measurement of outcomes and variables

Clinical effectiveness outcomes included ischemic stroke, MI, and their composite. Ischemic stroke events were identified as a primary diagnosis of inpatient claims using *ICD-10-CM* code I63 (except I63.1, I63.4, and I63.6; see Additional file 1). One hospitalization for ischemic stroke was considered one stroke event. MI was measured as a primary diagnosis of inpatient claims using *ICD-10-CM* code I21 or I22 (see Additional file 1). One hospitalization for MI was considered one MI event. An ischemic stroke or MI event was defined as the composite of ischemic stroke and MI [14]. Incidence rates and time to event occurrence were evaluated (events could be incident or recurrent).

HCRU outcomes included ASCVD-related and all-cause outpatient visits, hospitalizations, and emergency room (ER) visits. Cost outcomes included ASCVD-related and all-cause hospitalization costs and total medical costs. Total medical costs included medical costs from outpatient visits, hospitalizations, and ER visits. ASCVD-related HCRU and cost outcomes were measured using *ICD-10-CM* codes in the primary or secondary position (see Additional file 1). All-cause HCRU and medical costs included costs for any diagnosis, including ASCVD. The Optum CDM reports an estimated cost that is standardized based on a resource-based relative value scale derived from observed costs paid by the insurer, rather than the original paid amount.

### Statistical methods

Descriptive statistics were presented for all outcomes and covariates in the OW GLP-1 RA and DPP-4i groups. Counts and frequencies were used for categorical variables and means and standard deviations for continuous variables. Costs were reported as per person per month (PPPM) and adjusted to the year 2021. Incidence of first (or recurrent) stroke or MI in the follow-up (for those without and with a prior history of stroke/MI, respectively) was reported as number of events per 1000 person-years.

To reduce the observed selection bias between the 2 groups, inverse probability of treatment weighting (IPTW) [15] using average treatment effect weights was derived by conducting a logistic regression with the following variables: age, sex, race/ethnicity, geographic region, index year, Charlson Comorbidity Index, diabetes complications severity index, type of ASCVD history, comorbidities, glucose-lowering therapy, HbA1c, body mass index (BMI), and number of all-cause hospitalizations 60 days prior to the index date. For baseline characteristics, descriptive statistics were reported with and without IPTW. Standardized mean differences (SMDs) were presented. SMD < 10% was considered not significantly different for baseline characteristics. Weighted descriptive statistics were reported for all outcomes. For time-to-event outcomes, weighted cumulative incidence curves were generated and log-rank tests and Cox proportional-hazards (Cox-PH) regressions were conducted (proportional-hazards assumptions were met in these models). For HCRU and costs, generalized linear models with quasi-Poisson distribution and log-link function were used.

Interaction and stratified analyses were also conducted for the following variables: with and without history of ischemic stroke or MI, and end of follow-up before and after March 1, 2020, for HCRU and cost outcomes (to test the impact of COVID-19 on HCRU and costs). Sensitivity analyses excluding exenatide OW or including the prescriber type in weighting were also conducted. To assess residual unmeasured bias, negative control outcomes (ie, breast cancer and prostate cancer) and E-values were also examined [16]. To reduce bias due to informative censoring, inverse probability of censoring weighting (IPCW) was applied to assess clinical outcomes. To allow for a longer follow-up time, additional sensitivity analyses were conducted with the index date selection window restricted to between 2018 and 2020. Finally, to provide a complementary perspective, intention-to-treat (ITT) analyses were also performed for clinical outcomes.

## Results

Before weighting, the study included 26,430 OW GLP-1 RA users and 39,858 DPP-4i users (Additional file 3). After weighting, the sample sizes were 25,287 and 39,684, respectively. The average follow-up time was similar between the two groups (11.3 ± 9.3 months for OW GLP-1 RA; 11.3 ± 8.7 months for DPP-4i). After IPTW weighting, there were no significant differences in baseline characteristics, including use of other glucose-lowering treatment regimens, between the OW GLP-1 RA and DPP-4i groups (Additional file 2). The overall population had a mean age of 70 years and was nearly evenly split between males and females. Approximately 4 in 5 individuals were Medicare Advantage enrollees. Approximately 15% of people had a history of ischemic stroke and 15% had a history of MI. On average, individuals had 4.1 and 5.4 years of ASCVD and T2D history, respectively. In the OW GLP-1 RA group, most patients (62.9%) were taking dulaglutide while the remaining patients were taking once-weekly semaglutide (26.5%) or once-weekly exenatide (10.6%). In the DPP-4i group, most patients (67.3%) were on sitagliptin while the remaining patients were on linagliptin (27.1%), saxagliptin (4.8%), or alogliptin (0.8%).

### Clinical effectiveness outcomes

**Fig. 1:**
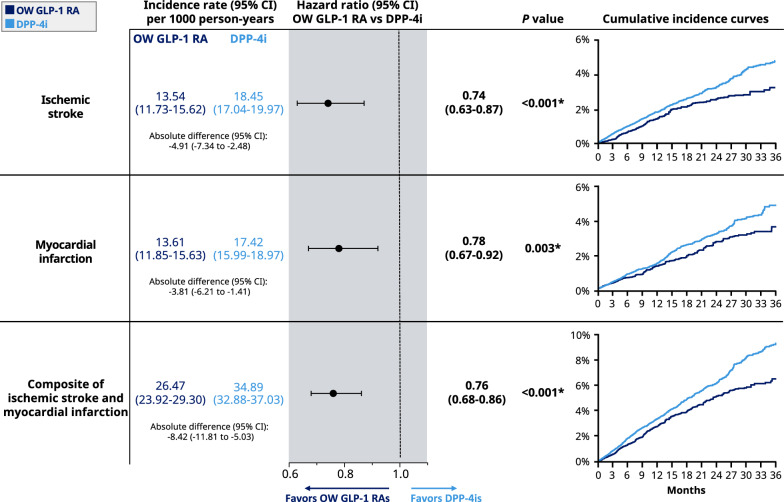
Clinical outcomes in adults with T2D and ASCVD on OW GLP-1 RAs or DPP-4is. Weighted incidence rates, hazard ratios, and cumulative incidence curves of clinical outcomes comparing OW GLP-1 RAs with DPP-4is among adults with T2D and ASCVD. *Indicates statistical significance (*P* < 0.05). *ASCVD* atherosclerotic cardiovascular disease, DPP-4is, dipeptidyl peptidase-4 inhibitors, *GLP-1 RAs* glucagon-like peptide-1 receptor agonists, *OW* once-weekly, *T2D* type 2 diabetes

Figure 1 displays the weighted incidence rates, hazard ratios (HRs), and cumulative incidence curves for clinical outcomes. Cox-PH regression results showed that compared with DPP-4is, OW GLP-1 RAs were associated with a 26% lower risk of ischemic stroke (HR [95% CI] 0.74 [0.63–0.87]). Additionally, OW GLP-1 RA users had a 22% lower risk of MI (HR [95% CI] 0.78 [0.67–0.92]) and a 24% lower risk of the composite of ischemic stroke or MI (HR [95% CI] 0.76 [0.68–0.86]) than did DPP-4i users. Incidence rates for stroke, MI, and their composite were − 4.91, − 3.81, and − 8.42 per 1000 person-years lower, respectively, in the OW GLP-1 RA group compared with the DPP-4i group. Similar trends were observed in the cumulative incidence curves for these clinical outcomes (Fig. 1).

### Health care resource utilization and cost outcomes

**Fig. 2:**
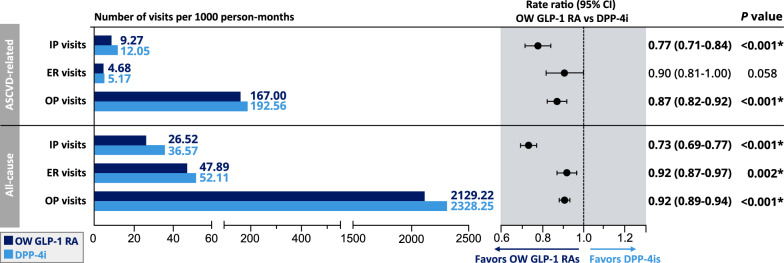
HCRU in adults with T2D and ASCVD on OW GLP-1 RAs or DPP-4is. Weighted descriptive statistics and rate ratios of HCRU comparing OW GLP-1 RAs with DPP-4is among adults with T2D and ASCVD. *Indicates statistical significance (*P* < 0.05). *ASCVD* atherosclerotic cardiovascular disease, *DPP-4is* dipeptidyl peptidase-4 inhibitors, *ER* emergency room, *GLP-1 RAs* glucagon-like peptide-1 receptor agonists, *HCRU* health care resource utilization, *IP* inpatient, *OP* outpatient, *OW* once-weekly, *T2D* type 2 diabetes

Figure 2 depicts the weighted HCRU descriptive statistics and rate ratios (RRs) in the OW GLP-1 RA group vs the DPP-4i group. OW GLP-1 RA users had fewer ASCVD-related and all-cause hospitalizations, ER visits, and outpatient visits. Regression models revealed significant differences in ASCVD-related hospitalizations and outpatient visits and all-cause hospitalizations, ER visits, and outpatient visits. Specifically, compared with DPP-4is, OW GLP-1 RAs were associated with 23% and 13% fewer ASCVD-related hospitalizations and outpatient visits, respectively. OW GLP-1 RA users also had 27%, 8%, and 8% fewer all-cause hospitalizations, ER visits, and outpatient visits, respectively, than did DPP-4i users.

**Fig. 3:**
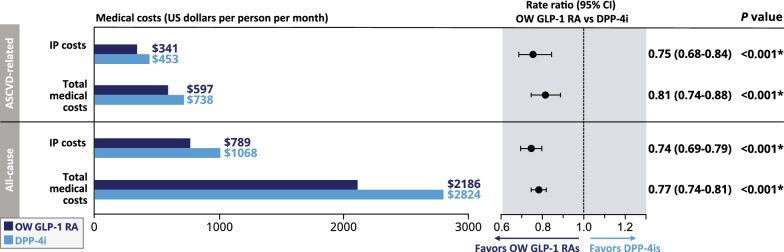
Medical costs in adults with T2D and ASCVD on OW GLP-1 RAs or DPP-4is. Weighted descriptive statistics and rate ratios of medical costs comparing OW GLP-1 RAs with DPP-4is among adults with T2D and ASCVD. *Indicates statistical significance (*P* < 0.05). *ASCVD* atherosclerotic cardiovascular disease, *DPP-4is* dipeptidyl peptidase-4 inhibitors, *GLP-1 RAs* glucagon-like peptide-1 receptor agonists, *IP* inpatient, *OW* once-weekly, *T2D* type 2 diabetes

Figure 3 shows the weighted descriptive statistics and RRs for medical costs in the OW GLP-1 RA and DPP-4i groups. Compared with DPP-4i users, OW GLP-1 RA users had 25% lower ASCVD-related hospitalization costs (RR [95% CI] 0.75 [0.68–0.84]) and 19% lower ASCVD-related total medical costs (RR [95% CI] 0.81 [0.74–0.88]). Average ASCVD-related hospitalization costs were $341 PPPM (OW GLP-1 RA) vs $453 PPPM (DPP-4i; absolute difference [95% CI] − $111 [− $153 to − $70] PPPM), and average ASCVD-related total medical costs were $597 PPPM (OW GLP-1 RA) vs $738 PPPM (DPP-4i; absolute difference [95% CI] − $141 [− $197 to − $85] PPPM). Compared with DPP-4is, OW GLP-1 RAs were associated with 26% lower all-cause hospitalization costs (RR [95% CI] 0.74 [0.69–0.79]) and 23% lower all-cause total medical costs (RR [95% CI] 0.77 [0.74–0.81]). All-cause hospitalization costs were $789 PPPM (OW GLP-1 RA) vs $1068 PPPM (DPP-4i; absolute difference [95% CI] − $279 [− $341 to − $217] PPPM), and all-cause total medical costs were $2186 PPPM (OW GLP-1 RA) vs $2824 PPPM (DPP-4i; absolute difference [95% CI] − $638 [− $753 to − $523] PPPM).

### Stratified and sensitivity analyses

After excluding exenatide, additional clinical risk reductions were identified in the OW GLP-1 RA group vs the DPP-4i group (Additional file 4). Similar trends were observed for HCRU and cost outcomes. Specifically, OW GLP-1 RAs (dulaglutide and semaglutide OW) were associated with 11% fewer ASCVD-related ER visits vs DPP-4is (HR [95% CI] 0.89 [0.80–0.99]; *P* = 0.032) (Additional file 5).

Results of sensitivity analyses including the prescriber type in IPTW were very similar to those of the main analyses (Additional file 6 and Additional file 7). The only major difference was that, after balancing on prescriber type, a significant reduction in ASCVD-related ER visits was identified in the OW GLP-1 RA group compared with the DPP-4i group (HR [95% CI] 0.90 [0.81–1.00]; *P* = 0.041).

No significant interactions were identified between stroke history and time to occurrence of stroke (*P* = 0.601) or between MI history and time to occurrence of MI (*P* = 0.794) between the two groups. There were trends toward decreased HCRU and costs in both groups among those who had an overlap with the COVID-19 pandemic compared with those who did not. However, no significant interactions were identified for HCRU and cost outcomes, except for a marginally significant interaction with all-cause hospitalizations (*P* = 0.050; data not shown). Analyses of negative control outcomes (ie, breast cancer and prostate cancer) showed no significant association. The E-value associated with the stroke outcome, representing the minimal strength of association an unmeasured confounder must have with stroke in the follow-up and treatment selection to change the result of the study, is 2.07 (upper confidence interval: 1.58).

Results generated from an analysis restricting the index date selection window to between 2018 and 2020 mirror the results from the main analysis (Additional file 8). OW GLP-1 RAs were significantly associated with a 27%, 20%, and 23% lower risk of ischemic stroke, MI, and their composite, respectively, compared to DPP-4is (all *P* < 0.05). The overall conclusions from the IPCW analysis are also similar to those of the main analysis (Additional file 9). With potentially informative censoring taken into consideration, IPCW analysis showed larger risk reductions for all clinical outcomes. More specifically, the IPCW analysis revealed a 31% reduction in risk of stroke, a 27% reduction in risk of MI, and a 29% reduction in risk of their composite in OW GLP-1 RA users compared with DPP-4i users (all *P* < 0.05). ITT analyses revealed smaller treatment differences between the two groups as compared to the main, per-protocol analysis (Additional file 10), which may be primarily driven by the 44.4% of patients who were not persistent with the index drug.

## Discussion

In a large sample of US adults with T2D and established ASCVD, treatment with OW GLP-1 RAs was associated with decreased risk of ischemic stroke and MI and fewer ASCVD-related and all-cause HCRU and medical costs compared with DPP-4is. The samples investigated were adequately sized to detect differences between groups, and individuals were followed for a relatively long time. The study was also designed and conducted to alleviate potential confounding and bias.

Several large, randomized trials have compared cardiovascular outcomes in people treated with OW GLP-1 RAs vs placebo. In the SUSTAIN 6 trial (semaglutide vs placebo), non-fatal stroke occurred in significantly fewer individuals in the semaglutide group (HR 0.61) [17]. Similar findings were observed in the REWIND trial (dulaglutide vs placebo) for both non-fatal stroke (HR 0.76) and non-fatal MI (HR 0.96) [18, 19]. The EXSCEL trial showed directionally similar efficacy results for exenatide OW [20, 21]. A recent meta-analysis of all GLP-1 RAs (including OW and once-daily versions) [22] assessing outcomes across CVOTs found that GLP-1 RAs reduced 3-point MACE by 14%, including a 10% reduction in MI risk and a 17% reduction in stroke risk. Upon exclusion of data from the ELIXA trial, which was conducted in a post-acute coronary syndrome population and used lixisenatide, known to be the least effective GLP-1 RA, risk reductions improved for all outcomes [22]. The HRs reported for stroke and MI in the present study are marginally lower than those reported in the above meta-analysis. Additionally, the sensitivity analysis excluding exenatide OW (which may have a limited effect on the cardiovascular outcomes of interest [20]) revealed lower HRs than did our original analyses including all three GLP-1 RA agents. Of note, the design of this study differed in that it compared OW GLP-1 RAs with DPP-4is and included some population differences (eg, including adults of all ages with T2D and ASCVD).

In real-world studies, GLP-1 RAs have consistently been associated with reduced cardiovascular risk in people with T2D [23–28], including reduced incidence of stroke (HRs between 0.65 and 0.74) and MI (HRs between 0.63 and 0.86). However, it is important to note that these studies were conducted in different countries and populations from this study. While previous studies have assessed cardiovascular outcomes in people with T2D and have largely assessed the older generation of GLP-1 RAs, the present study is among the first to examine cardiovascular outcomes in a large US population with T2D and ASCVD and those treated with the newer generation of OW GLP-1 RAs (eg, dulaglutide, semaglutide). However, as shown in clinical trials and in this study, these GLP-1 RAs may not be equivalent in terms of cardiovascular outcomes.

The mechanisms underlying the cardiovascular benefits of GLP-1 RAs are not fully known. However, these mechanisms may relate to a combination of effects on weight, blood pressure, glucose and lipid levels, inflammation, and potential effects on other factors promoting atherothrombosis [5].

Despite guideline recommendations and consistent evidence of cardiovascular risk reduction (including from the present study), DPP-4i use remains prevalent while the adoption of GLP-1 RAs is suboptimal among people with T2D and ASCVD [29], implying the need for a change in clinical practice. It is important that clinicians involved in the care of those with T2D and ASCVD consider or advise the use of GLP-1 RAs as well as other agents proven to lower cardiovascular risk (eg, SGLT-2 inhibitors, antihypertensive agents, lipid-lowering drugs, and antiplatelet agents where indicated) to reduce future atherothrombotic events. Compelling real-world evidence, such as the data provided in this study, supports clinical trial results and provides further evidence that could promote greater adoption of GLP-1 RAs into practice. In this study, the effect size identified for reduction in stroke risk (HR 0.74) is comparable to or exceeds that of meta-analyses investigating the effects of lowering blood pressure (RR 0.73), treating hyperlipidemia (RR 0.79), and other interventions in people with T2D [30], highlighting the benefits that can be achieved by adding GLP-1 RAs to antihyperglycemic regimens.

This study has several limitations. As an observational study, it is limited to assessments of associations and not causality. Additionally, the database used in this study includes predominantly commercial and Medicare Advantage plan enrollees, therefore caution is needed when applying the conclusions from this study to other populations. Finally, potential measurement errors (eg, misclassified billing codes), missing data, unavailability of information of date and cause of death, and use of imputed average cost data (rather than actual paid costs) may further limit the interpretation or applicability of these findings.

## Conclusion

Results from a large US administrative claims database revealed that, compared with the use of a DPP-4i, OW GLP-1 RA use among people with T2D and established ASCVD is associated with 26% lower ischemic stroke risk and 22% lower MI risk, along with significant reductions in ASCVD-related hospitalizations and outpatient visits; all-cause hospitalizations, ER visits, and outpatient visits; and ASCVD-related and all-cause hospitalization costs and total medical costs. This real-world evidence supports the findings from CVOTs and guideline recommendations [1, 6, 7] involving this class of glucose-lowering medications.

### Supplementary Information

**Additional file 1: **ASCVD Code List.

**Additional file 2: **All Unweighted and Weighted Baseline Characteristics Among Adults With T2D and ASCVD.

**Additional file 3: **Patient attrition flow chart.

**Additional file 4: **Weighted Outcomes for Ischemic Stroke, MI, and Their Composite Between OW GLP-1 RA (Excluding Exenatide OW) and DPP-4i Initiators Who Had T2D and Established ASCVD.

**Additional file 5: **Weighted HCRU and Cost Outcomes Between OW GLP-1 RA (Excluding Exenatide) and DPP-4i Initiators Who Had T2D and Established ASCVD.

**Additional file 6: **Weighted Outcomes for Ischemic Stroke, MI, and Their Composite Between OW GLP-1 RA and DPP-4i Initiators Who Had T2D and Established ASCVD, Including Prescriber's Type in Weighting.

**Additional file 7: **Weighted HCRU and Cost Outcomes Between OW GLP-1 RA and DPP-4i Initiators Who Had T2D and Established ASCVD, Including Prescriber's Type in Weighting.

**Additional file 8: **Clinical Outcomes in Adults with T2D and ASCVD on OW GLP-1 RAs or DPP-4is (Index Selection Window Between 2018 and 2020).

**Additional file 9: **IPCW Analysis of Clinical Outcomes in Adults With T2D and ASCVD on OW GLP-1 RAs or DPP-4is.

**Additional file 10: **ITT Analysis of Clinical Outcomes in Adults With T2D and ASCVD on OW GLP-1 RAs or DPP-4is.

## Data Availability

The Optum Clinformatics^®^ Data Mart was commercially licensed from the data vendor. Restrictions apply to the availability of these data, which were used under license of this study.
