# Supplementary material for: Once-weekly glucagon-like peptide-1 receptor agonists vs dipeptidyl peptidase-4 inhibitors: cardiovascular effects in people with diabetes and cardiovascular disease
Source: Cardiovasc Diabetol. 2023 Nov 20;22:319. doi: 10.1186/s12933-023-02051-8 (PMC10662529; doi:10.1186/s12933-023-02051-8)
Supplement: Supplementary file 1 — Additional file 1: ASCVD Code List. [file 12933_2023_2051_MOESM1_ESM.docx]

**Additional File 1. ASCVD Code List**

| **Diagnosis category** | ***ICD-9-CM*** | ***ICD-10-CM*** |
| --- | --- | --- |
| Ischemic stroke | 434 (except 434.1) | I63 (except I63.1, I63.4, I63.6) |
| Transient ischemic attack | 435 | G45 |
| Other atherosclerotic cerebrovascular disease | 433, 436, 437.0, 437.1, 438 | I65, I66, I67.2, I67.81 I67.82, I69 (except I69.0, I69.1, I69.2) |
| Myocardial infarction | 410 | I21, I22 |
| Other coronary heart disease | 412, 429.79, 411, 413, 414 (except 414.1) | I23, I24, I20, I25 (except I25.3, I25.4) |
| Peripheral arterial disease | 440, 443.8, 443.9, 444, 445 | I70, I73.9, I74, I75 |

ASCVD, atherosclerotic cardiovascular disease; *ICD-9-CM, International Classification of Diseases, Ninth Revision, Clinical Modification*; *ICD-10-CM, International Classification of Diseases, Tenth Revision, Clinical Modification*.

Note: *ICD-10-CM* codes of Z86.73 (Personal history of transient ischemic attack, and cerebral infarction without residual deficit) and Z86.74 (Personal history of sudden cardiac arrest) were also added to identify history of ASCVD. The code list was adapted from the 2018 Pantalone et al. ASCVD code set. [Pantalone KM, Misra-Hebert AD, Hobbs TM, et al. Antidiabetic treatment patterns and specialty care utilization among patients with type 2 diabetes and cardiovascular disease. *Cardiovasc Diabetol.* 2018;17(1):54. doi:10.1186/s12933-018-0699-7]
