# Supplementary material for: Once-weekly glucagon-like peptide-1 receptor agonists vs dipeptidyl peptidase-4 inhibitors: cardiovascular effects in people with diabetes and cardiovascular disease
Source: Cardiovasc Diabetol. 2023 Nov 20;22:319. doi: 10.1186/s12933-023-02051-8 (PMC10662529; doi:10.1186/s12933-023-02051-8)
Supplement: Supplementary file 2 — Additional file 2: All Unweighted and Weighted Baseline Characteristics Among Adults With T2D and ASCVD. [file 12933_2023_2051_MOESM2_ESM.docx]

**Additional File 2. All Unweighted and Weighted Baseline Characteristics Among Adults With T2D and ASCVD**

|  |  | **Unweighted** | | | | | **Weighted** | | | | |
| --- | --- | --- | --- | --- | --- | --- | --- | --- | --- | --- | --- |
|  |  | **DPP-4i (N=39,858)** | | **OW GLP-1 RA (N=26,430)** | |  | **DPP-4i (N=39,684)** | | **OW GLP-1 RA (N=25,287)** | |  |
| **Variable** | **Category** | **Mean** | **SD** | **Mean** | **SD** | **SMD** | **Mean** | **SD** | **Mean** | **SD** | **SMD** |
| Age, years | – | 72.67 | 9.75 | 66.53 | 10.08 | 0.619* | 70.26 | 10.32 | 69.68 | 10.19 | 0.057 |
| ASCVD, years | – | 4.20 | 3.80 | 4.07 | 3.81 | 0.034 | 4.14 | 3.80 | 4.12 | 3.78 | 0.005 |
| T2D, years | – | 5.40 | 4.13 | 5.42 | 4.30 | 0.005 | 5.34 | 4.14 | 5.47 | 4.24 | 0.029 |
| CCI score | – | 2.71 | 2.27 | 2.26 | 2.08 | 0.204* | 2.53 | 2.21 | 2.48 | 2.19 | 0.026 |
| DCSI score | – | 3.40 | 2.20 | 3.14 | 2.18 | 0.119* | 3.29 | 2.19 | 3.26 | 2.21 | 0.017 |
| HbA1c^a^ | – | 8.06 | 1.68 | 8.59 | 1.77 | 0.312* | 8.28 | 1.68 | 8.39 | 1.70 | 0.066 |
| BMI^b^, kg/m^2^ | – | 33.07 | 7.75 | 37.49 | 8.41 | 0.547* | 34.91 | 8.25 | 35.52 | 8.38 | 0.073 |
| Out-of-pocket Rx cost, USD | – | 721.87 | 819.93 | 878.14 | 992.05 | 0.172* | 753.72 | 823.55 | 814.52 | 932.05 | 0.069 |
| No. of ER visits | – | 0.76 | 1.74 | 0.74 | 2.00 | 0.012 | 0.74 | 1.76 | 0.73 | 1.91 | 0.006 |
| No. of IP visits | – | 0.69 | 1.64 | 0.46 | 1.22 | 0.163* | 0.60 | 1.49 | 0.55 | 1.42 | 0.033 |
| No. of IP visits (<60 days pre-index) | – | 0.23 | 0.78 | 0.08 | 0.40 | 0.245* | 0.17 | 0.66 | 0.14 | 0.57 | 0.055 |
| No. of IP visits (≥60 days pre-index) | – | 0.46 | 1.30 | 0.38 | 1.10 | 0.069 | 0.43 | 1.23 | 0.42 | 1.21 | 0.012 |
| **Variable** | **Category** | **n** | **%** | **n** | **%** | **SMD** | **n** | **%** | **n** | **%** | **SMD** |
| Had any claims of ASCVD at baseline (1 year prior to index) |  | 29,139 | 73.11 | 18,400 | 69.62 | 0.077 | 28,497 | 71.81 | 17,957 | 71.01 | 0.018 |
| Had any claims of ASCVD 90 days prior to index |  | 17,986 | 45.13 | 10,933 | 41.37 | 0.076 | 17,245 | 43.46 | 10,945 | 43.28 | 0.003 |
| Had any claims of ASCVD 30 days prior to index |  | 11,555 | 28.99 | 6531 | 24.71 | 0.097 | 10,913 | 27.5 | 6738 | 26.65 | 0.019 |
| Had any claims of stroke at baseline (1 year prior to index) |  | 3377 | 8.47 | 1742 | 6.59 | 0.071 | 3100 | 7.81 | 1866 | 7.38 | 0.016 |
| Had any claims of stroke 90 days prior to index |  | 1779 | 4.46 | 762 | 2.88 | 0.084 | 1562 | 3.94 | 869 | 3.43 | 0.027 |
| Had any claims of stroke 30 days prior to index |  | 1167 | 2.93 | 413 | 1.56 | 0.092 | 981 | 2.47 | 487 | 1.93 | 0.037 |
| Had any claims of MI at baseline (1 year prior to index) |  | 1971 | 4.95 | 1213 | 4.59 | 0.017 | 1860 | 4.69 | 1200 | 4.74 | 0.003 |
| Had any claims of MI 90 days prior to index |  | 997 | 2.50 | 503 | 1.90 | 0.041 | 874 | 2.20 | 575 | 2.27 | 0.005 |
| Had any claims of MI 30 days prior to index |  | 603 | 1.51 | 268 | 1.01 | 0.045 | 502 | 1.27 | 337 | 1.33 | 0.006 |
| Had any hospitalization for stroke at baseline (1 year prior to index) |  | 1219 | 3.06 | 487 | 1.84 | 0.079 | 1008 | 2.54 | 602 | 2.38 | 0.010 |
| Had any hospitalization for stroke 90 days prior to index) |  | 669 | 1.68 | 165 | 0.62 | 0.099 | 491 | 1.24 | 269 | 1.06 | 0.016 |
| Had any hospitalization for stroke 30 days prior to index |  | 379 | 0.95 | 61 | 0.23 | 0.094 | 267 | 0.67 | 114 | 0.45 | 0.030 |
| Had any hospitalization for MI at baseline (1 year prior to index) |  | 990 | 2.48 | 586 | 2.22 | 0.018 | 883 | 2.22 | 633 | 2.50 | 0.018 |
| Had any hospitalization for MI 90 days prior to index |  | 477 | 1.20 | 186 | 0.70 | 0.051 | 375 | 0.95 | 263 | 1.04 | 0.009 |
| Had any hospitalization for MI 30 days prior to index |  | 264 | 0.66 | 81 | 0.31 | 0.051 | 192 | 0.48 | 127 | 0.50 | 0.003 |
| **Variable** | **Category** | **n** | **%** | **n** | **%** | **SMD** | **n** | **%** | **n** | **%** | **SMD** |
| Age group | 18-44 y | 297 | 0.8 | 618 | 2.3 | 0.130* | 539 | 1.4 | 367 | 1.5 | 0.008 |
|  | 45-64 y | 6928 | 17.4 | 9651 | 36.5 | 0.442* | 9852 | 24.8 | 6628 | 26.2 | 0.032 |
|  | 65-79 y | 22,570 | 56.6 | 13,924 | 52.7 | 0.079 | 21,909 | 55.2 | 14,151 | 56.0 | 0.015 |
|  | ≥80 y | 10,063 | 25.3 | 2237 | 8.5 | 0.460* | 7384 | 18.6 | 4141 | 16.4 | 0.059 |
| Sex | F | 19,953 | 50.1 | 12,767 | 48.3 | 0.035 | 19,645 | 49.5 | 12,387 | 49.0 | 0.010 |
|  | M | 19,905 | 49.9 | 13,663 | 51.7 | 0.035 | 20,039 | 50.5 | 12,900 | 51.0 | 0.010 |
| Region | 0; Northeast | 4823 | 12.1 | 2491 | 9.4 | 0.086 | 4348 | 11.0 | 2656 | 10.5 | 0.015 |
|  | 1; South | 21,514 | 54.0 | 14,620 | 55.3 | 0.027 | 21,767 | 54.9 | 13,981 | 55.3 | 0.009 |
|  | 2; Midwest | 6417 | 16.1 | 4842 | 18.3 | 0.059 | 6671 | 16.8 | 4272 | 16.9 | 0.002 |
|  | 3; West | 7082 | 17.8 | 4462 | 16.9 | 0.023 | 6874 | 17.3 | 4360 | 17.2 | 0.002 |
|  | 4; Unknown | 22 | 0.1 | 15 | 0.1 | 0.001 | 24 | 0.1 | 18 | 0.1 | 0.005 |
| Payer type | COM | 5111 | 12.8 | 7397 | 28.0 | 0.383* | 7295 | 18.4 | 4940 | 19.5 | 0.029 |
|  | MCR | 34,747 | 87.2 | 19,033 | 72.0 | 0.383* | 32,389 | 81.6 | 20,347 | 80.5 | 0.029 |
| Plan type | EPO | 667 | 1.7 | 1094 | 4.1 | 0.147* | 1000 | 2.5 | 700 | 2.8 | 0.015 |
|  | HMO | 8968 | 22.5 | 5243 | 19.8 | 0.065 | 8593 | 21.7 | 5486 | 21.7 | 0.001 |
|  | IND | 265 | 0.7 | 133 | 0.5 | 0.021 | 229 | 0.6 | 139 | 0.6 | 0.004 |
|  | OTH | 24,760 | 62.1 | 13,692 | 51.8 | 0.209* | 23,087 | 58.2 | 14,425 | 57.0 | 0.023 |
|  | POS | 3433 | 8.6 | 5215 | 19.7 | 0.323* | 5071 | 12.8 | 3440 | 13.6 | 0.024 |
|  | PPO | 1765 | 4.4 | 1053 | 4.0 | 0.022 | 1705 | 4.3 | 1098 | 4.3 | 0.002 |
| Index year | 2018 | 11,683 | 29.3 | 4369 | 16.5 | 0.308* | 9759 | 24.6 | 5970 | 23.6 | 0.023 |
|  | 2019 | 11,297 | 28.3 | 7388 | 28.0 | 0.009 | 11,251 | 28.4 | 7192 | 28.4 | 0.002 |
|  | 2020 | 10,412 | 26.1 | 8414 | 31.8 | 0.126* | 11,172 | 28.2 | 7225 | 28.6 | 0.009 |
|  | 2021 | 6466 | 16.2 | 6259 | 23.7 | 0.187* | 7501 | 18.9 | 4900 | 19.4 | 0.012 |
| HbA1c | 0; <7% | 4626 | 11.6 | 2061 | 7.8 | 0.129* | 3938 | 9.9 | 2258 | 8.9 | 0.034 |
|  | 1; 7-<8% | 5692 | 14.3 | 2776 | 10.5 | 0.115* | 5043 | 12.7 | 3061 | 12.1 | 0.018 |
|  | 2; 8-<9% | 4289 | 10.8 | 2863 | 10.8 | 0.002 | 4362 | 11.0 | 2842 | 11.2 | 0.008 |
|  | 3; 9%+ | 4393 | 11.0 | 4344 | 16.4 | 0.158* | 5303 | 13.4 | 3575 | 14.1 | 0.023 |
|  | 4; Unknown | 20,858 | 52.3 | 14,386 | 54.4 | 0.042 | 21,038 | 53.0 | 13,551 | 53.6 | 0.012 |
| BMI | 0; <25 | 1603 | 4.0 | 319 | 1.2 | 0.177* | 1166 | 2.9 | 617 | 2.4 | 0.031 |
|  | 1; 25-<30 | 3361 | 8.4 | 1432 | 5.4 | 0.119* | 2874 | 7.2 | 1771 | 7.0 | 0.009 |
|  | 2; 30-<35 | 3804 | 9.5 | 2668 | 10.1 | 0.019 | 3874 | 9.8 | 2502 | 9.9 | 0.004 |
|  | 3; 35-<40 | 2333 | 5.9 | 2479 | 9.4 | 0.133* | 2854 | 7.2 | 1903 | 7.5 | 0.013 |
|  | 4; 40+ | 2112 | 5.3 | 3317 | 12.6 | 0.256* | 3272 | 8.3 | 2215 | 8.8 | 0.018 |
|  | 5; Unknown | 26,645 | 66.9 | 16,215 | 61.4 | 0.115* | 25,643 | 64.6 | 16,280 | 64.4 | 0.005 |
| Race/ethnicity | 0; White | 21,936 | 55.0 | 16,479 | 62.4 | 0.149* | 22,988 | 57.9 | 14,866 | 58.8 | 0.017 |
|  | 1; Black | 6496 | 16.3 | 4256 | 16.1 | 0.005 | 6457 | 16.3 | 4137 | 16.4 | 0.002 |
|  | 2; Hispanic | 7566 | 19.0 | 3886 | 14.7 | 0.115* | 6838 | 17.2 | 4211 | 16.7 | 0.015 |
|  | 3; Asian | 1970 | 4.9 | 581 | 2.2 | 0.148* | 1519 | 3.8 | 854 | 3.4 | 0.024 |
|  | 4; Unknown | 1890 | 4.7 | 1228 | 4.7 | 0.005 | 1882 | 4.7 | 1220 | 4.8 | 0.004 |
| Number of glucose-lowering therapies | 0 | 3937 | 9.9 | 1652 | 6.3 | 0.134* | 3260 | 8.2 | 2032 | 8.0 | 0.006 |
|  | 1 | 16,921 | 42.5 | 8905 | 33.7 | 0.181* | 15,416 | 38.9 | 9232 | 36.5 | 0.048 |
|  | 2 | 14,294 | 35.9 | 10,102 | 38.2 | 0.049 | 14,648 | 36.9 | 9669 | 38.2 | 0.027 |
|  | 3+ | 4706 | 11.8 | 5771 | 21.8 | 0.271* | 6360 | 16.0 | 4354 | 17.2 | 0.032 |
| Glucose-lowering therapy use | Metformin | 28,044 | 70.4 | 18,060 | 68.3 | 0.044 | 27,641 | 69.7 | 17,630 | 69.7 | 0.001 |
|  | SU | 16,648 | 41.8 | 9436 | 35.7 | 0.125* | 15,893 | 40.1 | 10,234 | 40.5 | 0.009 |
|  | TZD | 3010 | 7.6 | 2263 | 8.6 | 0.037 | 3216 | 8.1 | 2127 | 8.4 | 0.011 |
|  | SGLT-2 | 3785 | 9.5 | 5435 | 20.6 | 0.313* | 5599 | 14.1 | 3819 | 15.1 | 0.028 |
|  | Insulin | 7946 | 19.9 | 11,888 | 45.0 | 0.555* | 11,792 | 29.7 | 8019 | 31.7 | 0.043 |
|  | AGI | 191 | 0.5 | 117 | 0.4 | 0.005 | 188 | 0.5 | 128 | 0.5 | 0.005 |
|  | MEG | 619 | 1.6 | 276 | 1.0 | 0.045 | 538 | 1.4 | 333 | 1.3 | 0.003 |
| Other medication use | Anticoagulants | 6037 | 15.2 | 3536 | 13.4 | 0.051 | 5789 | 14.6 | 3454 | 13.7 | 0.027 |
|  | Antihypertensives | 36,784 | 92.3 | 24,286 | 91.9 | 0.015 | 36,536 | 92.1 | 23,207 | 91.8 | 0.011 |
|  | Antiplatelets | 7252 | 18.2 | 4822 | 18.2 | 0.001 | 7212 | 18.2 | 4693 | 18.6 | 0.010 |
|  | Misc hyperlipidemic | 5656 | 14.2 | 4335 | 16.4 | 0.061 | 5921 | 14.9 | 3852 | 15.2 | 0.009 |
|  | PCSK9 | 146 | 0.4 | 228 | 0.9 | 0.064 | 178 | 0.5 | 179 | 0.7 | 0.034 |
|  | Statins | 32,277 | 81.0 | 21,731 | 82.2 | 0.032 | 32,274 | 81.3 | 20,627 | 81.6 | 0.006 |
| Other comorbidities | Atrial fibrillation | 6669 | 16.7 | 3478 | 13.2 | 0.100* | 6075 | 15.3 | 3646 | 14.4 | 0.025 |
|  | Alcohol use disorder | 767 | 1.9 | 474 | 1.8 | 0.010 | 781 | 2.0 | 435 | 1.7 | 0.018 |
|  | Anxiety | 6681 | 16.8 | 5114 | 19.4 | 0.067 | 6959 | 17.5 | 4553 | 18.0 | 0.012 |
|  | Depression | 9456 | 23.7 | 7145 | 27.0 | 0.076 | 9766 | 24.6 | 6551 | 25.9 | 0.030 |
|  | Hyperlipidemia | 35,306 | 88.6 | 23,633 | 89.4 | 0.027 | 35,167 | 88.6 | 22,539 | 89.1 | 0.016 |
|  | Hypertension | 37,304 | 93.6 | 24,534 | 92.8 | 0.030 | 36,990 | 93.2 | 23,540 | 93.1 | 0.005 |
|  | Obesity | 14,950 | 37.5 | 14,857 | 56.2 | 0.382* | 17,837 | 45.0 | 11,737 | 46.4 | 0.029 |
|  | Smoking | 4598 | 11.5 | 3556 | 13.5 | 0.058 | 4998 | 12.6 | 3033 | 12.0 | 0.018 |
|  | Chronic heart failure | 9961 | 25.0 | 6026 | 22.8 | 0.051 | 9538 | 24.0 | 5970 | 23.6 | 0.010 |
|  | Cancer | 4883 | 12.3 | 2431 | 9.2 | 0.099 | 4401 | 11.1 | 2683 | 10.6 | 0.015 |
|  | CKD | 15,924 | 40.0 | 8086 | 30.6 | 0.197* | 14,403 | 36.3 | 8896 | 35.2 | 0.023 |
| ASCVD-related procedures | CABG | 1273 | 3.2 | 774 | 2.9 | 0.015 | 1217 | 3.1 | 775 | 3.1 | 0.000 |
|  | PCI | 899 | 2.3 | 720 | 2.7 | 0.030 | 881 | 2.2 | 691 | 2.7 | 0.033 |
| Type of ASCVD | MI | 5840 | 14.7 | 3842 | 14.5 | 0.003 | 5767 | 14.5 | 3672 | 14.5 | 0.000 |
|  | Ischemic stroke | 6545 | 16.4 | 3602 | 13.6 | 0.078 | 6047 | 15.2 | 3745 | 14.8 | 0.012 |
|  | PAD | 21,438 | 53.8 | 12,565 | 47.6 | 0.125* | 20,373 | 51.4 | 12,827 | 50.7 | 0.012 |
|  | TIA | 5106 | 12.8 | 2864 | 10.8 | 0.061 | 4780 | 12.1 | 2936 | 11.6 | 0.013 |
|  | Other atherosclerotic cerebrovascular disease | 14,350 | 36.0 | 7942 | 30.1 | 0.127* | 13,444 | 33.9 | 8304 | 32.9 | 0.022 |
|  | Other CHD | 26,059 | 65.4 | 17,800 | 67.4 | 0.042 | 26,167 | 66.0 | 16,837 | 66.6 | 0.014 |
| **CCI details** | **Category** | **n** | **%** | **n** | **%** | **SMD** | **n** | **%** | **n** | **%** | **SMD** |
| CCI category | 0-1 | 13,874 | 34.8 | 11,432 | 43.3 | 0.174* | 15,140 | 38.2 | 9846 | 38.9 | 0.016 |
|  | 2 | 7083 | 17.8 | 4908 | 18.6 | 0.021 | 7119 | 17.9 | 4708 | 18.6 | 0.018 |
|  | 3 | 6450 | 16.2 | 3846 | 14.6 | 0.045 | 6180 | 15.6 | 3852 | 15.2 | 0.009 |
|  | 4 | 4978 | 12.5 | 2717 | 10.3 | 0.070 | 4641 | 11.7 | 2864 | 11.3 | 0.012 |
|  | 5 | 3276 | 8.2 | 1636 | 6.2 | 0.079 | 2946 | 7.4 | 1821 | 7.2 | 0.009 |
|  | ≥6 | 4197 | 10.5 | 1891 | 7.2 | 0.119* | 3657 | 9.2 | 2196 | 8.7 | 0.019 |
| AIDS | 6 | 140 | 0.4 | 140 | 0.5 | 0.027 | 161 | 0.4 | 113 | 0.5 | 0.006 |
| Cerebrovascular | 1 | 9716 | 24.4 | 5287 | 20.0 | 0.105* | 9018 | 22.7 | 5605 | 22.2 | 0.013 |
| Dementia | 1 | 3333 | 8.4 | 1083 | 4.1 | 0.177* | 2675 | 6.7 | 1618 | 6.4 | 0.014 |
| Diabetes | 1 | 13,418 | 33.7 | 9216 | 34.9 | 0.025 | 13,541 | 34.1 | 8740 | 34.6 | 0.009 |
|  | 2 | 26,412 | 66.3 | 17,201 | 65.1 | 0.025 | 26,117 | 65.8 | 16,526 | 65.4 | 0.010 |
| Heart | 1 | 9961 | 25.0 | 6026 | 22.8 | 0.051 | 9538 | 24.0 | 5970 | 23.6 | 0.010 |
| Hemiparaplegia | 2 | 1112 | 2.8 | 534 | 2.0 | 0.050 | 984 | 2.5 | 575 | 2.3 | 0.014 |
| Liver | 1 | 3668 | 9.2 | 2753 | 10.4 | 0.041 | 3835 | 9.7 | 2494 | 9.9 | 0.007 |
|  | 3 | 291 | 0.7 | 212 | 0.8 | 0.008 | 291 | 0.7 | 181 | 0.7 | 0.001 |
| Malignancy | 2 | 4864 | 12.2 | 2417 | 9.1 | 0.099 | 4385 | 11.1 | 2664 | 10.5 | 0.017 |
| Metastatic | 6 | 545 | 1.4 | 203 | 0.8 | 0.058 | 463 | 1.2 | 282 | 1.1 | 0.005 |
| Myocardial | 1 | 4922 | 12.4 | 3197 | 12.1 | 0.008 | 4831 | 12.2 | 3076 | 12.2 | 0.000 |
| Peptic ulcer | 1 | 749 | 1.9 | 390 | 1.5 | 0.031 | 694 | 1.8 | 428 | 1.7 | 0.004 |
| Peripheral-vascular | 1 | 13,722 | 34.4 | 7550 | 28.6 | 0.126* | 12,788 | 32.2 | 8007 | 31.7 | 0.012 |
| Pulmonary | 1 | 11,068 | 27.8 | 7483 | 28.3 | 0.012 | 11,087 | 27.9 | 7039 | 27.8 | 0.002 |
| Renal | 2 | 15,924 | 40.0 | 8086 | 30.6 | 0.197* | 14,403 | 36.3 | 8896 | 35.2 | 0.023 |
| Rheumatic | 1 | 1967 | 4.9 | 1280 | 4.8 | 0.004 | 1936 | 4.9 | 1219 | 4.8 | 0.003 |
| **DCSI details** | **Category** | **n** | **%** | **n** | **%** | **SMD** | **n** | **%** | **n** | **%** | **SMD** |
| DCSI category | 0 | 3645 | 9.1 | 2928 | 11.1 | 0.064 | 3867 | 9.8 | 2661 | 10.5 | 0.026 |
|  | 1 | 4728 | 11.9 | 3767 | 14.3 | 0.071 | 5103 | 12.9 | 3348 | 13.2 | 0.011 |
|  | 2 | 7103 | 17.8 | 4925 | 18.6 | 0.021 | 7296 | 18.4 | 4500 | 17.8 | 0.015 |
|  | 3 | 6193 | 15.5 | 4219 | 16.0 | 0.012 | 6228 | 15.7 | 3999 | 15.8 | 0.003 |
|  | 4 | 6241 | 15.7 | 3698 | 14.0 | 0.047 | 5970 | 15.0 | 3682 | 14.6 | 0.014 |
|  | 5 | 4923 | 12.4 | 2960 | 11.2 | 0.036 | 4689 | 11.8 | 2983 | 11.8 | 0.001 |
|  | ≥6 | 7025 | 17.6 | 3933 | 14.9 | 0.074 | 6530 | 16.5 | 4114 | 16.3 | 0.005 |
| Cardiovascular | 1 | 10,640 | 26.7 | 7115 | 26.9 | 0.005 | 10,619 | 26.8 | 6757 | 26.7 | 0.001 |
|  | 2 | 16,396 | 41.1 | 10,016 | 37.9 | 0.066 | 15,772 | 39.8 | 9941 | 39.3 | 0.009 |
| Cerebrovascular | 1 | 657 | 1.7 | 425 | 1.6 | 0.003 | 653 | 1.6 | 432 | 1.7 | 0.005 |
|  | 2 | 7174 | 18.0 | 3932 | 14.9 | 0.084 | 6663 | 16.8 | 4119 | 16.3 | 0.013 |
| Metabolic | 2 | 118 | 0.3 | 81 | 0.3 | 0.002 | 116 | 0.3 | 77 | 0.3 | 0.002 |
| Nephropathy | 1 | 2027 | 5.1 | 1744 | 6.6 | 0.065 | 2230 | 5.6 | 1442 | 5.7 | 0.004 |
|  | 2 | 16,308 | 40.9 | 8331 | 31.5 | 0.196* | 14,772 | 37.2 | 9144 | 36.2 | 0.022 |
| Neuropathy | 1 | 16,031 | 40.2 | 11,650 | 44.1 | 0.078 | 16,592 | 41.8 | 10,597 | 41.9 | 0.002 |
| Peripheral-vascular | 1 | 11,077 | 27.8 | 6659 | 25.2 | 0.059 | 10,650 | 26.8 | 6733 | 26.6 | 0.005 |
|  | 2 | 2172 | 5.5 | 1653 | 6.3 | 0.034 | 2263 | 5.7 | 1478 | 5.8 | 0.006 |
| Retinopathy | 1 | 6452 | 16.2 | 4214 | 15.9 | 0.007 | 6362 | 16.0 | 4030 | 15.9 | 0.003 |
|  | 2 | 2174 | 5.5 | 1575 | 6.0 | 0.022 | 2236 | 5.6 | 1440 | 5.7 | 0.003 |

AGI, alpha-glucosidase inhibitor; ASCVD, atherosclerotic cardiovascular disease; BMI, body mass index; CABG, coronary artery bypass grafting; CCI, Charlson Comorbidity Index; CHD, coronary heart disease; CKD, chronic kidney disease; COM, commercial; DCSI, diabetes complication severity index; DPP-4i, dipeptidyl peptidase-4 inhibitor; EPO, exclusive provider organization; ER, emergency room; GLP-1 RA, glucagon-like peptide-1 receptor agonist; HbA1c, glycated hemoglobin; HMO, health maintenance organization; IND, individual; IP, inpatient; MCR, Medicare; MEG, meglitinide; MI, myocardial infarction; OTH, other; OW, once-weekly; PAD, peripheral arterial disease; PCI, percutaneous coronary intervention; PCSK9, proprotein convertase subtilisin/kexin type 9; POS, point of service; PPO, preferred provider organization; Rx, prescription; SGLT-2, sodium-glucose cotransporter 2 [inhibitor]; SMD, standardized mean difference; SU, sulfonylurea; T2D, type 2 diabetes; TIA, transient ischemic attack; TZD, thiazolidinedione.

*Indicates significant difference.

^a^HbA1c results reflect only those individuals for whom these data were available.

^b^BMI results reflect only those individuals for whom these data were available. Continuous BMI values were calculated as the midpoint of the value range for the corresponding BMI code. (For example, a value of 32.5 was used for the *ICD-10* code Z68.32, which includes BMI 32.0-32.9.)
