# Supplementary material for: Once-weekly glucagon-like peptide-1 receptor agonists vs dipeptidyl peptidase-4 inhibitors: cardiovascular effects in people with diabetes and cardiovascular disease
Source: Cardiovasc Diabetol. 2023 Nov 20;22:319. doi: 10.1186/s12933-023-02051-8 (PMC10662529; doi:10.1186/s12933-023-02051-8)
Supplement: Supplementary file 3 — Additional file 3: Patient attrition flow chart. [file 12933_2023_2051_MOESM3_ESM.docx]

**
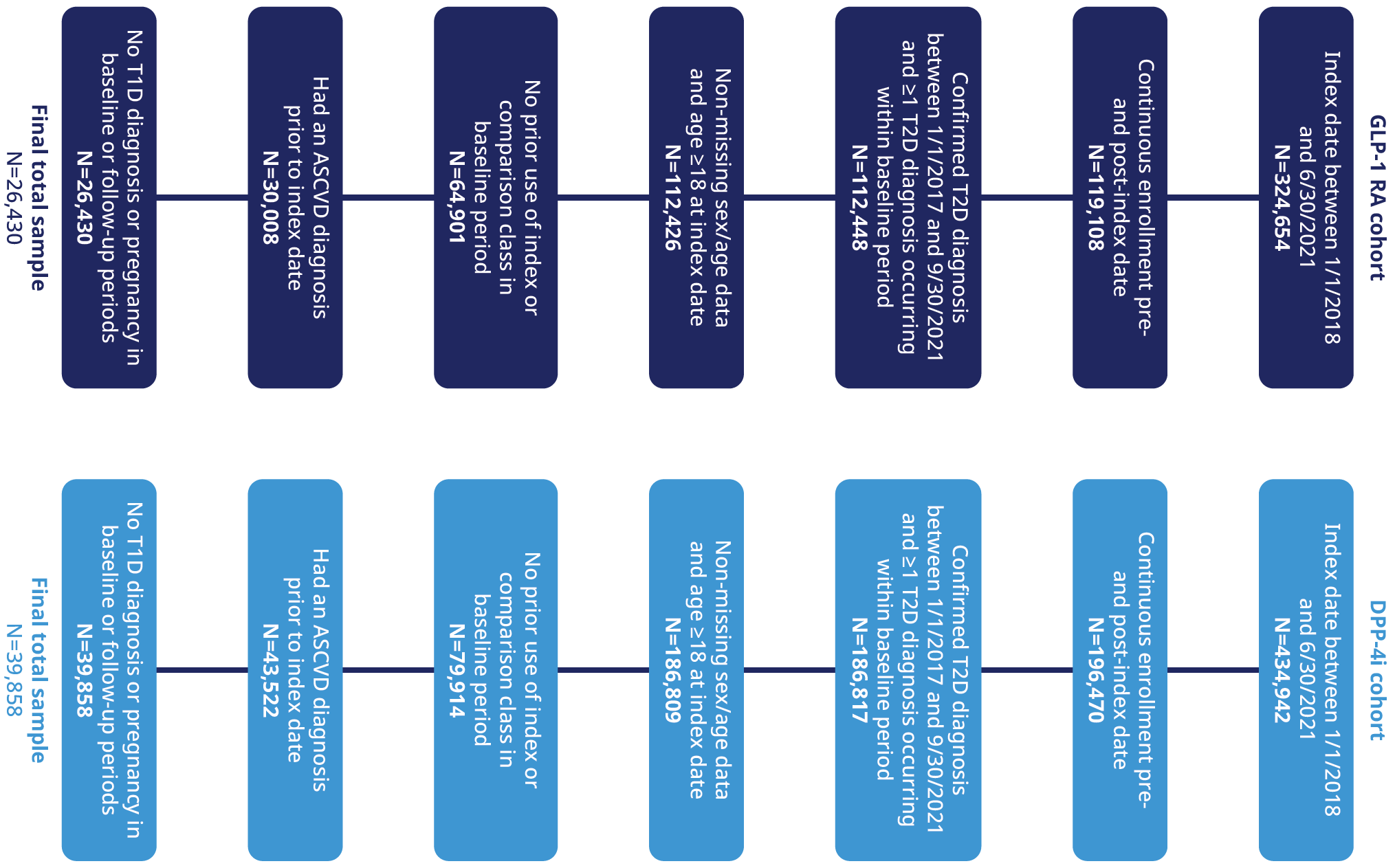
**

**Additional File 3. Patient attrition flow chart.** ASCVD, atherosclerotic cardiovascular disease; DPP-4i, dipeptidyl peptidase-4 inhibitor; GLP-1 RA, glucagon-like peptide-1 receptor agonist; T1D, type 1 diabetes; T2D, type 2 diabetes.
