# Supplementary material for: Once-weekly glucagon-like peptide-1 receptor agonists vs dipeptidyl peptidase-4 inhibitors: cardiovascular effects in people with diabetes and cardiovascular disease
Source: Cardiovasc Diabetol. 2023 Nov 20;22:319. doi: 10.1186/s12933-023-02051-8 (PMC10662529; doi:10.1186/s12933-023-02051-8)
Supplement: Supplementary file 4 — Additional file 4: Weighted Outcomes for Ischemic Stroke, MI, and Their Composite Between OW GLP-1 RA (Excluding Exenatide OW) and DPP-4i Initiators Who Had T2D and Established ASCVD. [file 12933_2023_2051_MOESM4_ESM.docx]

**Additional File 4. Weighted Outcomes for Ischemic Stroke, MI, and Their Composite Between OW GLP-1 RA (Excluding Exenatide OW) and DPP-4i Initiators Who Had T2D and Established ASCVD**

|  | **Non-exenatide OW GLP-1 RA** | **DPP-4i** | **Non-exenatide OW GLP-1 RA vs DPP-4i** | | **Non-exenatide OW GLP-1 RA vs DPP-4i** | |
| --- | --- | --- | --- | --- | --- | --- |
|  | **n=22,837** | **n=39,676** |  |  | **Cox proportional-hazards model** | |
|  | **Incidence rate (95% CI)**  **per 1000 person-years** | | **Incidence rate ratio (95% CI)** | ***P* value** | **Hazard ratio (95% CI)** | ***P* value** |
| **Ischemic stroke** | 13.16 (11.28-15.35) | 18.57 (17.17-20.09) | 0.71 (0.60-0.84) | **<0.001** | 0.71 (0.60-0.85) | **<0.001** |
| **MI** | 13.04 (11.20-15.17) | 17.43 (16.01-18.96) | 0.75 (0.63-0.89) | **0.001** | 0.75 (0.63-0.89) | **0.001** |
| **Composite of ischemic stroke and MI** | 25.47 (22.82-28.44) | 35.02 (33.02-37.14) | 0.73 (0.64-0.82) | **<0.001** | 0.73 (0.65-0.83) | **<0.001** |

ASCVD, atherosclerotic cardiovascular disease; DPP-4i, dipeptidyl peptidase-4 inhibitor; GLP-1 RA, glucagon-like peptide-1 receptor agonist; MI, myocardial infarction; OW, once-weekly; T2D, type 2 diabetes.
