# Supplementary material for: Once-weekly glucagon-like peptide-1 receptor agonists vs dipeptidyl peptidase-4 inhibitors: cardiovascular effects in people with diabetes and cardiovascular disease
Source: Cardiovasc Diabetol. 2023 Nov 20;22:319. doi: 10.1186/s12933-023-02051-8 (PMC10662529; doi:10.1186/s12933-023-02051-8)
Supplement: Supplementary file 5 — Additional file 5: Weighted HCRU and Cost Outcomes Between OW GLP-1 RA (Excluding Exenatide) and DPP-4i Initiators Who Had T2D and Established ASCVD. [file 12933_2023_2051_MOESM5_ESM.docx]

**Additional File 5. Weighted HCRU and Cost Outcomes Between OW GLP-1 RA (Excluding Exenatide) and DPP-4i Initiators Who Had T2D and Established ASCVD**

|  | **OW GLP-1 RA n=22, 837** | **DPP-4i n=39,676** | **OW GLP-1 RA vs DPP-4i** | |
| --- | --- | --- | --- | --- |
|  | **Incidence rate (95% CI)** | | **Rate ratio (95% CI)** | ***P* value** |
| *ASCVD-related HCRU, 1000 person-months* | | | | |
| **ASCVD-related ER visits** | 4.62 (4.22-5.06) | 5.20 (4.91-5.51) | 0.89 (0.80-0.99) | **0.032** |
| **ASCVD-related IP visits** | 9.11 (8.42-9.85) | 12.13 (11.64-12.63) | 0.75 (0.69-0.82) | **<0.001** |
| **ASCVD-related OP visits** | 168.62 (159.26-178.54) | 193.77 (187.36-200.40) | 0.87 (0.81-0.93) | **<0.001** |
| *All-cause HCRU, 1000 person-months* | | | | |
| **All-cause ER visits** | 47.08 (44.94-49.33) | 52.15 (50.63-53.71) | 0.90 (0.85-0.95) | **<0.001** |
| **All-cause IP visits** | 26.31 (24.96-27.73) | 36.84 (35.80-37.90) | 0.71 (0.67-0.76) | **<0.001** |
| **All-cause OP visits** | 2136.29 (2087.99-2185.71) | 2338.08 (2306.26-2370.33) | 0.91 (0.89-0.94) | **<0.001** |
| *Costs, US dollars PPPM* | | | | |
| **ASCVD-related IP costs** | 329 (299-363) | 454 (430-479) | 0.73 (0.65-0.81) | **<0.001** |
| **ASCVD-related total medical costs** | 582 (537-629) | 739 (708-771) | 0.79 (0.72-0.86) | **<0.001** |
| **All-cause IP costs** | 770 (723-820) | 1073 (1034-1112) | 0.72 (0.67-0.77) | **<0.001** |
| **All-cause total medical costs** | 2143 (2061-2229) | 2830 (2755-2909) | 0.76 (0.72-0.79) | **<0.001** |

ASCVD, atherosclerotic cardiovascular disease; DPP-4i, dipeptidyl peptidase-4 inhibitor; ER, emergency room; GLP-1 RA, glucagon-like peptide-1 receptor agonist; HCRU, health care resource utilization; IP, inpatient; OP, outpatient; OW, once-weekly; PPPM, per person per month; T2D, type 2 diabetes.
