# Supplementary material for: Once-weekly glucagon-like peptide-1 receptor agonists vs dipeptidyl peptidase-4 inhibitors: cardiovascular effects in people with diabetes and cardiovascular disease
Source: Cardiovasc Diabetol. 2023 Nov 20;22:319. doi: 10.1186/s12933-023-02051-8 (PMC10662529; doi:10.1186/s12933-023-02051-8)
Supplement: Supplementary file 6 — Additional file 6: Weighted Outcomes for Ischemic Stroke, MI, and Their Composite Between OW GLP-1 RA and DPP-4i Initiators Who Had T2D and Established ASCVD, Including Prescriber's Type in Weighting. [file 12933_2023_2051_MOESM6_ESM.docx]

**Additional File 6. Weighted Outcomes for Ischemic Stroke, MI, and Their Composite Between OW GLP-1 RA and DPP-4i Initiators Who Had T2D and Established ASCVD, Including Prescriber's Type in Weighting**

|  | **OW GLP-1 RA** | **DPP-4i** | **OW GLP-1 RA vs DPP-4i** | | **OW GLP-1 RA vs DPP-4i** | |
| --- | --- | --- | --- | --- | --- | --- |
|  | **n=25,327** | **n=39,577** |  |  | **Cox proportional-hazards model** | |
|  | **Incidence rate (95% CI)**  **per 1000 person-years** | | **Incidence rate ratio (95% CI)** | ***P* value** | **Hazard ratio (95% CI)** | ***P* value** |
| **Ischemic stroke** | 13.49 (11.70-15.55) | 18.51 (17.11-20.03) | 0.73 (0.62-0.86) | **<0.001** | 0.73 (0.62-0.86) | **<0.001** |
| **MI** | 13.60 (11.84-15.63) | 17.36 (15.92-18.92) | 0.78 (0.67-0.92) | **0.003** | 0.79 (0.67-0.92) | **0.004** |
| **Composite of ischemic stroke and MI** | 26.46 (23.92-29.28) | 34.93 (32.90-37.07) | 0.76 (0.67-0.52) | **<0.001** | 0.76 (0.68-0.85) | **<0.001** |

ASCVD, atherosclerotic cardiovascular disease; DPP-4i, dipeptidyl peptidase-4 inhibitor; GLP-1 RA, glucagon-like peptide-1 receptor agonist; MI, myocardial infarction; OW, once-weekly; T2D, type 2 diabetes.
