# Supplementary material for: Once-weekly glucagon-like peptide-1 receptor agonists vs dipeptidyl peptidase-4 inhibitors: cardiovascular effects in people with diabetes and cardiovascular disease
Source: Cardiovasc Diabetol. 2023 Nov 20;22:319. doi: 10.1186/s12933-023-02051-8 (PMC10662529; doi:10.1186/s12933-023-02051-8)
Supplement: Supplementary file 7 — Additional file 7: Weighted HCRU and Cost Outcomes Between OW GLP-1 RA and DPP-4i Initiators Who Had T2D and Established ASCVD, Including Prescriber's Type in Weighting. [file 12933_2023_2051_MOESM7_ESM.docx]

**Additional File 7. Weighted HCRU and Cost Outcomes Between OW GLP-1 RA and DPP-4i Initiators Who Had T2D and Established ASCVD, Including Prescriber's Type in Weighting**

|  | **OW GLP-1 RA n=22,837** | **DPP-4i n=39,676** | **OW GLP-1 RA vs DPP-4i** | |
| --- | --- | --- | --- | --- |
|  | **Incidence rate (95% CI)** | | **Rate ratio (95% CI)** | ***P* value** |
| *ASCVD-related HCRU, 1000 person-months* | | | | |
| **ASCVD-related ER visits** | 4.65 (4.27-5.08) | 5.19 (4.90-5.50) | 0.90 (0.81-1.00) | **0.041** |
| **ASCVD-related IP visits** | 9.25 (8.58-9.98) | 12.04 (11.55-12.55) | 0.77 (0.71-0.84) | **<0.001** |
| **ASCVD-related OP visits** | 166.21 (157.53-175.38) | 192.35 (186.07-198.85) | 0.86 (0.81-0.92) | **<0.001** |
| *All-cause HCRU, 1000 person-months* | | | | |
| **All-cause ER visits** | 47.72 (45.72-49.82) | 52.35 (50.79-53.96) | 0.91 (0.87-0.96) | **<0.001** |
| **All-cause IP visits** | 26.75 (25.44-28.13) | 36.54 (35.50-37.62) | 0.73 (0.69-0.78) | **<0.001** |
| **All-cause OP visits** | 2125.73 (2080.58-2171.87) | 2337.50 (2304.92-2370.54) | 0.91 (0.89-0.93) | **<0.001** |
| *Costs, US dollars PPPM* | | | | |
| **ASCVD-related IP costs** | 340 (308-375) | 454 (429-480) | 0.75 (0.67-0.84) | **<0.001** |
| **ASCVD-related total medical costs** | 595 (550-644) | 740 (708-773) | 0.81 (0.74-0.88) | **<0.001** |
| **All-cause IP costs** | 795 (747-846) | 1071 (1031-1111) | 0.74 (0.69-0.80) | **<0.001** |
| **All-cause total medical costs** | 2192 (2106-2280) | 2839 (2759-2921) | 0.77 (0.74-0.81) | **<0.001** |

ASCVD, atherosclerotic cardiovascular disease; DPP-4i, dipeptidyl peptidase-4 inhibitor; ER, emergency room; GLP-1 RA, glucagon-like peptide-1 receptor agonist; HCRU, health care resource utilization; IP, inpatient; OP, outpatient; OW, once-weekly; PPPM, per person per month; T2D, type 2 diabetes.
