# Supplementary material for: Once-weekly glucagon-like peptide-1 receptor agonists vs dipeptidyl peptidase-4 inhibitors: cardiovascular effects in people with diabetes and cardiovascular disease
Source: Cardiovasc Diabetol. 2023 Nov 20;22:319. doi: 10.1186/s12933-023-02051-8 (PMC10662529; doi:10.1186/s12933-023-02051-8)
Supplement: Supplementary file 8 — Additional file 8: Clinical Outcomes in Adults with T2D and ASCVD on OW GLP-1 RAs or DPP-4is (Index Selection Window Between 2018 and 2020). [file 12933_2023_2051_MOESM8_ESM.docx]

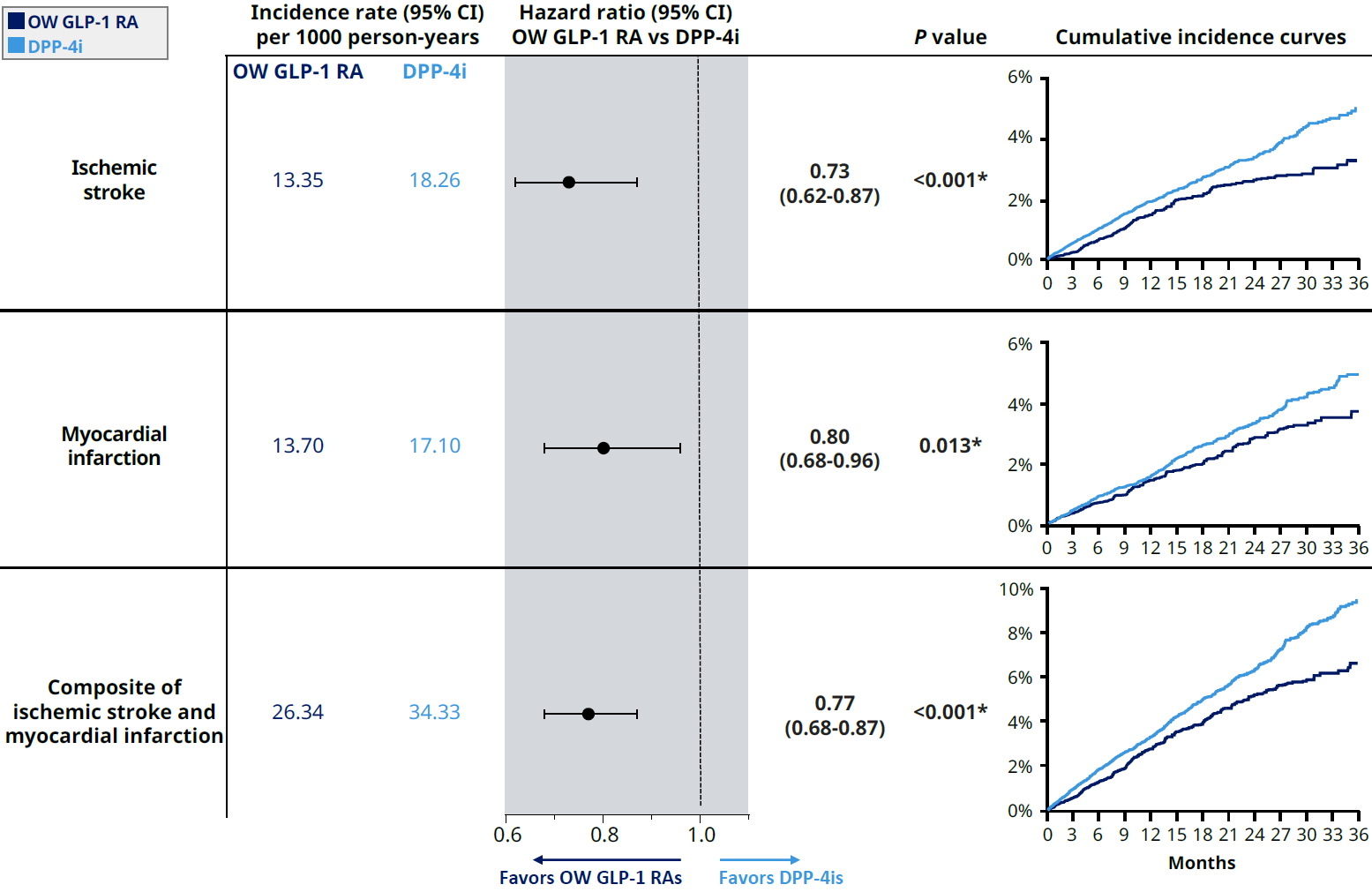


**Additional File 8. Clinical Outcomes in Adults with T2D and ASCVD on OW GLP-1 RAs or DPP-4is (Index Selection Window Between 2018 and 2020).** Weighted incidence rates, hazard ratios, and cumulative incidence curves of clinical outcomes comparing OW GLP-1 RAs with DPP-4is among adults with T2D and ASCVD with index date between 2018 and 2020. *Indicates statistical significance (*P*<0.05). ASCVD, atherosclerotic cardiovascular disease; DPP-4is, dipeptidyl peptidase-4 inhibitors; GLP-1 RAs, glucagon-like peptide-1 receptor agonists; OW, once-weekly; T2D, type 2 diabetes.
