# Supplementary material for: Once-weekly glucagon-like peptide-1 receptor agonists vs dipeptidyl peptidase-4 inhibitors: cardiovascular effects in people with diabetes and cardiovascular disease
Source: Cardiovasc Diabetol. 2023 Nov 20;22:319. doi: 10.1186/s12933-023-02051-8 (PMC10662529; doi:10.1186/s12933-023-02051-8)
Supplement: Supplementary file 10 — Additional file 10: ITT Analysis of Clinical Outcomes in Adults With T2D and ASCVD on OW GLP-1 RAs or DPP-4is. [file 12933_2023_2051_MOESM10_ESM.docx]

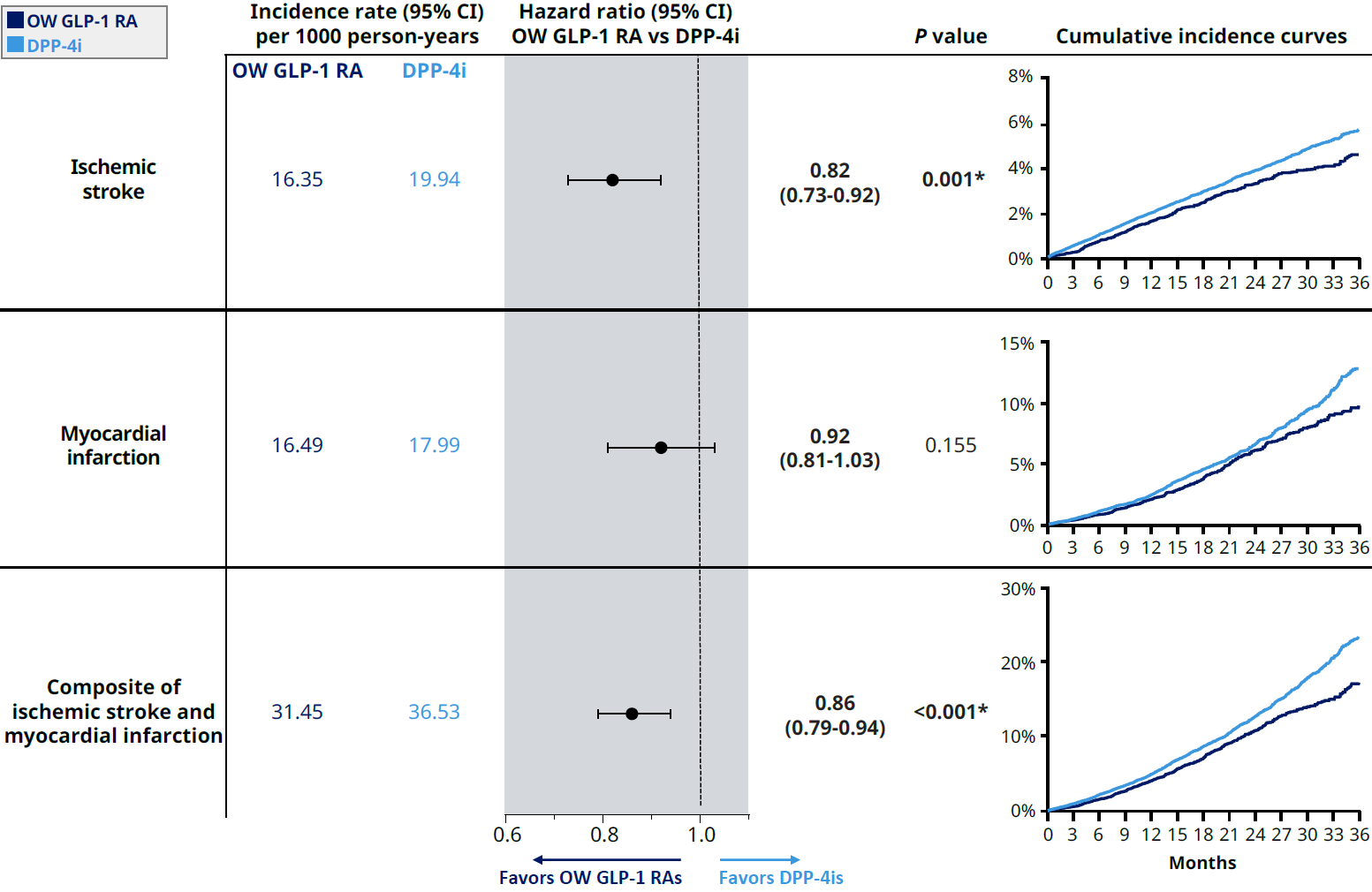


**Additional File 10. ITT Analysis of Clinical Outcomes in Adults With T2D and ASCVD on OW GLP-1 RAs or DPP-4is.** ITT analysis of weighted incidence rates, hazard ratios, and cumulative incidence curves of clinical outcomes comparing OW GLP-1 RAs with DPP-4is among adults with T2D and ASCVD. *Indicates statistical significance (*P*<0.05). ASCVD, atherosclerotic cardiovascular disease; DPP-4is, dipeptidyl peptidase-4 inhibitors; GLP-1 RAs, glucagon-like peptide-1 receptor agonists; ITT, intention-to-treat; OW, once-weekly; T2D, type 2 diabetes.
